# Interaction within and between cortical networks subserving multisensory learning and its reorganization due to musical expertise

**DOI:** 10.1038/s41598-022-12158-9

**Published:** 2022-05-12

**Authors:** Evangelos Paraskevopoulos, Nikolas Chalas, Alexandra Anagnostopoulou, Panagiotis D. Bamidis

**Affiliations:** 1 Department of Psychology, University of Cyprus, P.O. Box 20537, CY 1678 Nicosia, Cyprus; 2 Institute for Biomagnetism and Biosignalanalysis, University of Münster, Münster, Germany; 3 School of Medicine, Faculty of Health Sciences, Aristotle University of Thessaloniki, Thessaloniki, Greece

**Keywords:** Cognitive neuroscience, Sensory processing, Cortex

## Abstract

Recent advancements in the field of network science allow us to quantify inter-network information exchange and model the interaction within and between task-defined states of large-scale networks. Here, we modeled the inter- and intra- network interactions related to multisensory statistical learning. To this aim, we implemented a multifeatured statistical learning paradigm and measured evoked magnetoencephalographic responses to estimate task-defined state of functional connectivity based on cortical phase interaction. Each network state represented the whole-brain network processing modality-specific (auditory, visual and audiovisual) statistical learning irregularities embedded within a multisensory stimulation stream. The way by which domain-specific expertise re-organizes the interaction between the networks was investigated by a comparison of musicians and non-musicians. Between the modality-specific network states, the estimated connectivity quantified the characteristics of a supramodal mechanism supporting the identification of statistical irregularities that are compartmentalized and applied in the identification of uni-modal irregularities embedded within multisensory stimuli. Expertise-related re-organization was expressed by an increase of intra- and a decrease of inter-network connectivity, showing increased compartmentalization.

## Introduction

The traditional approach in our understanding of multisensory integration highlights the modular and hierarchical structure of multisensory processes^1^, while recent views emphasized the emergence of multisensory perception as the result of a dynamic interplay of large-scale networks^2^. This latter view is supported by studies showing early multisensory interactions^3,4^, direct connections between unisensory areas^5^, and feed-forward and feedback processing of multisensory convergence^6^. Synergistic processing of multiple sensory stimuli requires a modular integrational pathway: each stimulus is initially processed by the corresponding sensory modality, while its output is subsequently integrated with the output of other sensory systems. On the other hand, the processing of supramodal representations actuates directly across senses at a network level^7^, affecting unisensory areas at a later stage^8^; hence treating multisensory input processing as a single modality.

Each of these processing routes might comprise several hierarchically organized -and potentially- overlapping large-scale cortical networks. In this case, specific cortical regions can contribute to multiple of these networks, differentiating their functionality according to the task-defined state of the system^9^. For instance, the intraparietal lobule, prefrontal, superior, and middle temporal regions comprise a set of important hubs for multisensory processing networks^10^, but their role in different modality-specific network states is yet to be defined. Recent advancements in the field of multilayer network science allow us to quantify inter-network information exchange^11^ and quantify the interaction within and between task-specific states of large-scale networks^12^. This approach may contribute to our understanding of how the interaction of task-specific states of cortical networks may reflect the hierarchy of the cognitive processes composing multisensory integration.

Communication between cortical areas contributing to multisensory processing is considered to be facilitated by increased neuronal synchronization^13^ and subserved by oscillatory processes^14^. Triggering the underlying processes of cortical re-organization, multisensory learning is one of the most efficient neuroplasticity drivers^15,16^. An example of multisensory learning is the extraction of transitional probabilities embedded in multisensory stimulation streams, a process often so-called *statistical learning*^17^. It has been proposed that statistical learning subserves segmenting of sensory information streams into chunks, based on their transitional probabilities, and thus facilitates subsequent decoding of structured incoming information^18^. Frost et al.^19^ proposed that statistical learning comprises a supramodal learning mechanism, respecting restrictions determined by modality-specific networks. To this end, a set of brain regions (inferior frontal gyrus, caudate, thalamus, hippocampus) may form a domain-general network while other a domain-specific (superior temporal gyrus and inferior parietal lobule for the auditory domain and cuneus and fusiform gyrus for the visual domain). Hence, a unified supra-modal mechanism is compartmentalized and employed in sections of different sensory modalities. Nonetheless, this categorization has not been found after a direct comparison of the functionality of these regions.

One way to examine domain specificity is to investigate how domain-specific expertise modifies the neuronal processes related to this functionality^20^. Expert musicians show enhanced statistical learning abilities, as well as a re-organization of the neural underpinnings supporting statistical learning^21^. A recent study by Paraskevopoulos et al.^22^ introduced a modular multisensory paradigm to investigate audiovisual statistical learning that allowed the disentangling of independent uni- and multi-modal contributions, the latter being localized in inferior frontal and medial temporal regions. Neuroplastic effects related to musical expertise in this study included enhanced connectivity between the right temporal lobe and left inferior frontal gyrus and top-down modulation of their activity by the pre-SMA and the STG. Taking into account that those regions have been found to be involved in the processing of both uni- and multi-sensory statistical learning, their possible role in coordinating inter-network communication remains unexplored.

Here, we quantify the inter- and intra-network interaction (as indexed by local communication between the nodes of a network vs. global communication between networks) of task-specific states of large-scale networks subserving multisensory integration. To this aim, we employed a multisensory statistical learning paradigm via magnetoencephalography. The paradigm incorporated 3 parallel streams of stimulation with independent transitional probabilities: an auditory, a visual, and an audiovisual one, combined in audiovisual stimuli. Differences in the inter- and intra- network communication associated with music expertise are quantified via a comparison of musicians to non-musicians. We hypothesize that regions of the functional networks representing supra-modal processing (i.e., inferior frontal, pre-SMA, intra-parietal lobule, and medial temporal sources) will show similar connectivity profiles during the identification of modality-specific irregularities embedded within multisensory stimuli, displaying meaningful information transfer between the different networks. On the contrary, regions representing the processing of modality-specific irregularities (i.e., posterior superior temporal sources, secondary visual sources) will show functional independence. Thereby we aim to identify the supra-modal mechanism of statistical learning and the role of each region in the interaction of the different network states. In addition, we hypothesized that domain-specific expertise (i.e., musical training) modify both inter- and intra- network interactions re-organizing the information transfer structure, resulting in significant differences between musicians and non-musicians.

## Results

### Modality specific cortical networks supporting the processing of statistical irregularities

**Figure 1:**
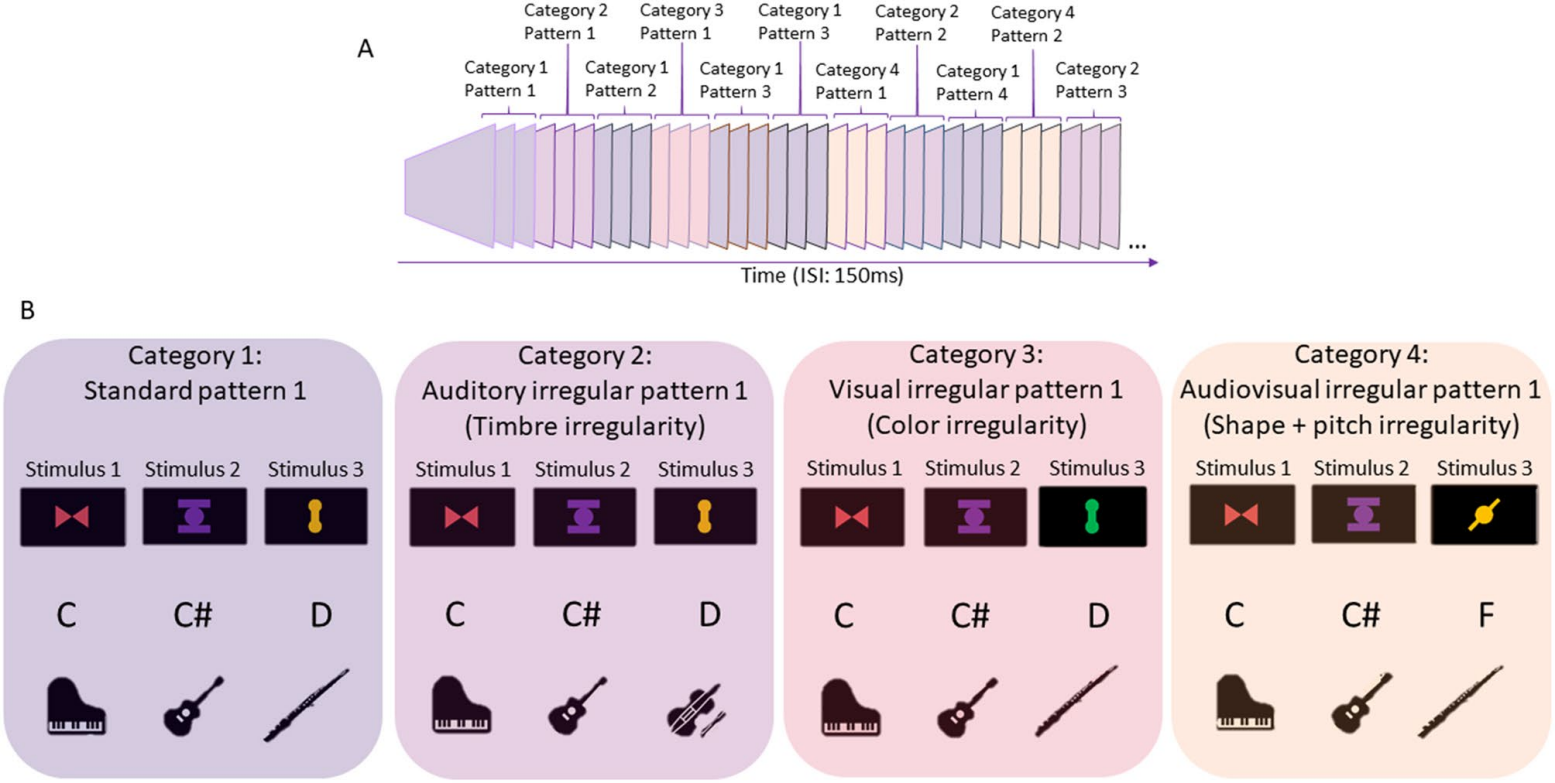
Example description of stimulation stream and stimuli categories: (**A**): Stimuli are presented in a continuous stream with constant ISI. Standard stimuli patterns and irregular ones of each category are randomly interleaved. (**B**): The first block presents one random stimulus pattern from the category of standard stimuli; all characteristics (shape, color, pitch, timbre) are paired according to the transitional probabilities of the multisensory stream. Second block represents the category of auditory irregular stimulus; timbre is altered by using the timbre of the first stimulus of another standard pattern, thus violating transitional probabilities of the auditory part of the stream. All other characteristics remain correct. Third block represents the category of visual irregular stimulus, color is altered by using the color of the first stimulus of another standard pattern, violating transitional probabilities of the visual part of the stream. All other characteristics remain correct. Fourth block represents the category of audiovisual incongruent stimulus; shape and pitch are altered, using the shape and pitch of the first stimulus of another standard pattern, thus violating transitional probabilities, while all other characteristics remain correct.

To identify cortical networks supporting the processing of modality-specific statistical irregularities within a multisensory stimulation stream, we measured the magnetoecephalographic responses of 25 subjects (12 musicians and 13 non-musicians) that were exposed to a multisensory stimulation stream. Each stimulus characteristic (pitch, timbre, shape, and color) followed independent transitional probabilities, unifying thereby 4 parallel statistical learning streams. The latter incorporated stimuli patterns that either followed the transitional probabilities for all stimulus characteristics and thus were considered as standard, or the last stimulus of the pattern violated the probabilities with respect to one or two of the characteristics producing modality-specific irregularities. Specifically, if the transitional probabilities of timbre were violated, the irregularity was auditory; if the color was violated, the irregularity was visual; if pitch and shape were violated concurrently, the irregularity was audiovisual (Fig. 1).

**Figure 2:**
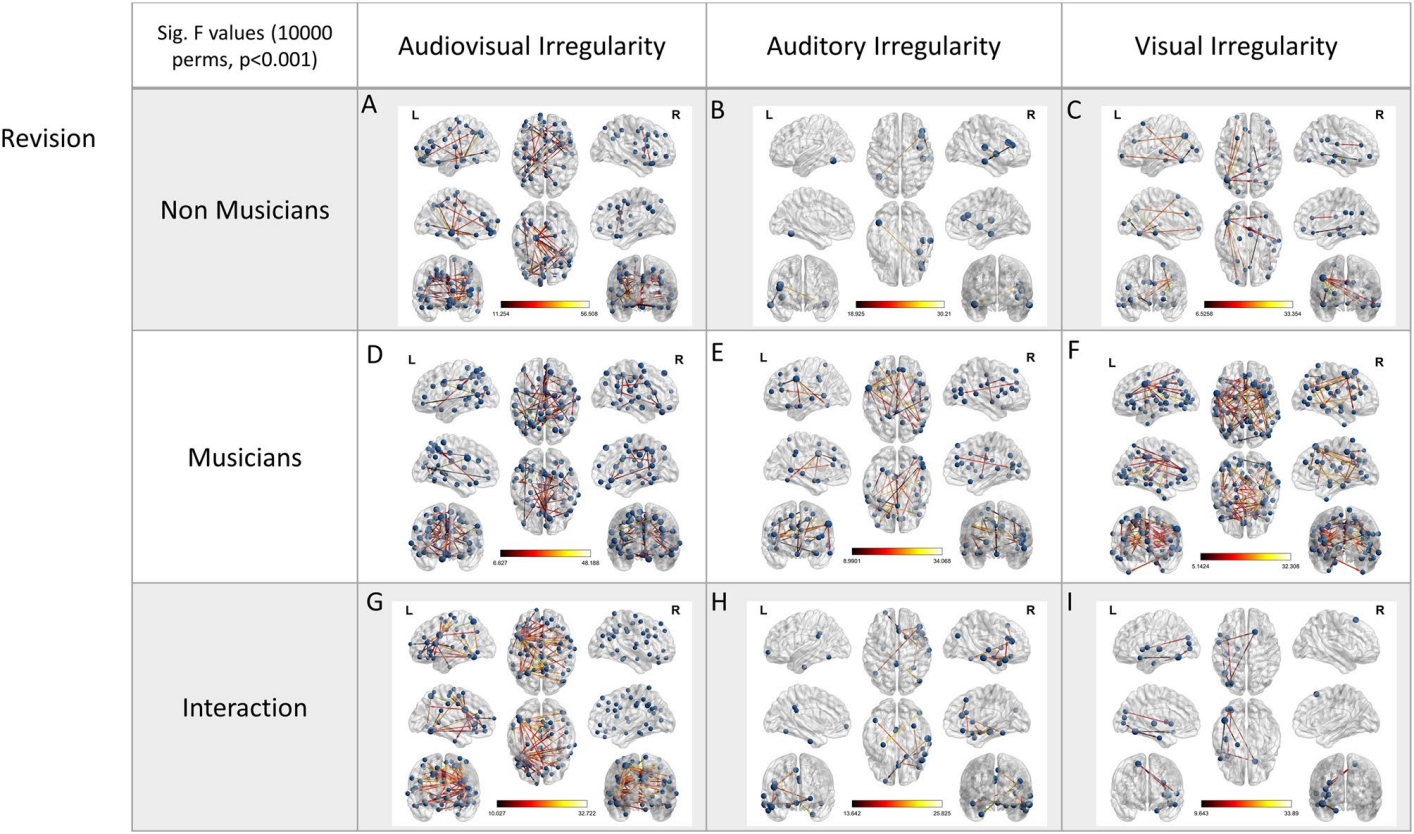
Modality-specific networks supporting the identification of statistical learning irregularities and music related neuroplasticity: Statistical parametric maps of the significant networks for the regular ≠ irregular stimulus comparison for each modality, presented in a tabular form. First row contains results of condition (statistically regular vs. statistically irregular stimulus) non-musicians, for each irregularity detection modality, second row presents results of musicians, while the third row presents the 2 × 2 interaction of condition (regular vs. irregular stimulus) × group (musicians vs. non-musicians), and hence, the effect of expertise related re-organization, for each irregularity detection modality. The color scale indicates F values, while node strength is represented by node size. Networks presented are significant at *p* < 0.001, FDR corrected, using 10.000 permutations.

Τo account for the fact that multisensory integration is strongly subserved by phase interaction of distributed cortical regions^23^, we estimated the functional connectivity of each single trial’s source reconstructed activity via Phase Transfer Entropy (PTE). We used the 360 sources of the atlas proposed by Glasser et al. 2016 to balance spatial resolution and functional specificity of the reconstructed activity. The adjacency matrices of each subject, for each deviant condition, were then statistically compared to the adjacency matrices of the responses to the standard pattern, to model the functional network supporting the identification of statistical learning irregularities for each modality (auditory, visual and audiovisual) and each subject group. The re-organization of the corresponding network due to musical-expertise-related neuroplasticity was estimated by a statistical comparison of the functional connectivity of musicians and non-musicians. The results of this analysis are summarized in Fig. 2.

### Audiovisual irregularity, non-musicians (Fig. 2A)

The modeling of the network supporting the processing of audiovisual irregularities of non-musicians was based on t-tests comparing the PTE derived functional connectivity networks for the final stimulus of standard and audiovisual deviant patterns. This analysis identified a significant network (nodes: 52; edges: 41) connecting medial temporal, intraparietal, and frontal sources. The region with the greater role in the network as identified from node strength was the left presubiculum.

### Audiovisual irregularity, musicians (Fig. 2D)

The corresponding t-test for the audiovisual modality of musicians identified a significant network (nodes: 63; edges: 45) mainly comprising of temporal, intraparietal, and frontal regions. The area with the greater role in the network as identified from node strength was the left 33 prime.

### Audiovisual irregularity, group difference (Fig. 2G)

The interaction of the 2 × 2 mixed model ANOVA depicting the difference between musicians and non musicians in the network supporting the identification of statistical learning irregularities of audiovisual nature [i.e., factor group (musicians vs. non-musicians) × the factor condition (regular vs. audiovisually irregular)] identified a significant network (nodes: 67; edges: 67) comprising mostly of left medial temporal and inferior frontal regions. The region with the greatest role in the network is left IFJa (inferior frontal junction).

### Auditory modality, non-musicians (Fig. 2B)

The modeling of the network supporting the processing of auditory irregularities of non-musicians was based on t-tests comparing the PTE derived functional connectivity networks for the final stimulus of standard and auditory irregular patterns. This analysis identified a significant network (nodes: 6; edges: 3). As determined from node strength, the region with the greater role in the network was the left fusiform gyrus.

### Auditory modality, musicians (Fig. 2E)

The corresponding t-test for the auditory modality of musicians identified a significant network (nodes: 35; edges: 23) comprising mostly of connections from frontal and temporal regions. The region with the greater role in the network as identified from node strength was the left ventral area 6.

### Auditory modality, group difference (Fig. 2H)

The interaction of the 2 × 2 mixed model ANOVA depicting the difference between musicians and non musicians in the network supporting the identification of statistical learning irregularities of auditory nature identified a significant network (nodes: 18; edges: 13) comprising mostly of right frontotemporal regions. The region with the greater role in the network is right TE1m.

### Visual modality, non-musicians (Fig. 2C)

The corresponding test for the visual modality of non-musicians identified a significant network (nodes: 22; edges: 17) comprising of connections from inferior temporal, intraparietal and frontal areas. The region with the greatest role in the network, as identified from node strength, is the left PGs.

### Visual modality, musicians (Fig. 2F)

The network of musicians (nodes: 76; edges: 63) mainly consisted of connections following the dorsal pathway. The area with the greatest role in the network is the left 6vL.

### Visual modality, group difference (Fig. 2I)

The interaction of the 2 × 2 mixed model ANOVA depicting the difference between musicians and non-musicians in the network supporting the identification of statistical learning irregularities of visual nature identified a significant network (nodes: 7; edges: 10) comprising mostly of secondary visual, inferior temporal and inferior frontal regions. The region with the greatest role in the network, as identified from node strength, was the left fusiform gyrus. The significance level for all above-mentioned analyses was set to *p* < 0.001 corrected for multiple comparisons via false discovery rate (FDR) correction, with 10.000 permutations. Figure 2 presents the networks supporting modality-specific identification of statistical learning irregularities.

### Interaction within and between the modality-specific states

For modeling the inter- and intra- modal interactions underlying multisensory integration, we formed one graph for each subject, that included 4 different modality-specific network states (i.e., the cortical networks of the 4 different conditions: standard, auditory deviant, visual deviant, and audiovisual irregularity), and their interactions. Within each state, the graph included the adjacency matrices containing the z-score transformation of PTE-derived edges amongst the 360 regions of the HCP atlas. Between each state, the graph included the adjacency matrices containing the corresponding mutlilinks, that is, the significant edge-to-edge correlations between all different conditions’ networks in pairs. Statistical significance of the connectivity amongst the different sensory modalities was estimated via one-sample t-tests of each group’s hypergraph. The corresponding music-related neuroplasticity was estimated via an independent samples t-test comparing the hypergraphs of musicians to the ones of non-musicians.

**Figure 3:**
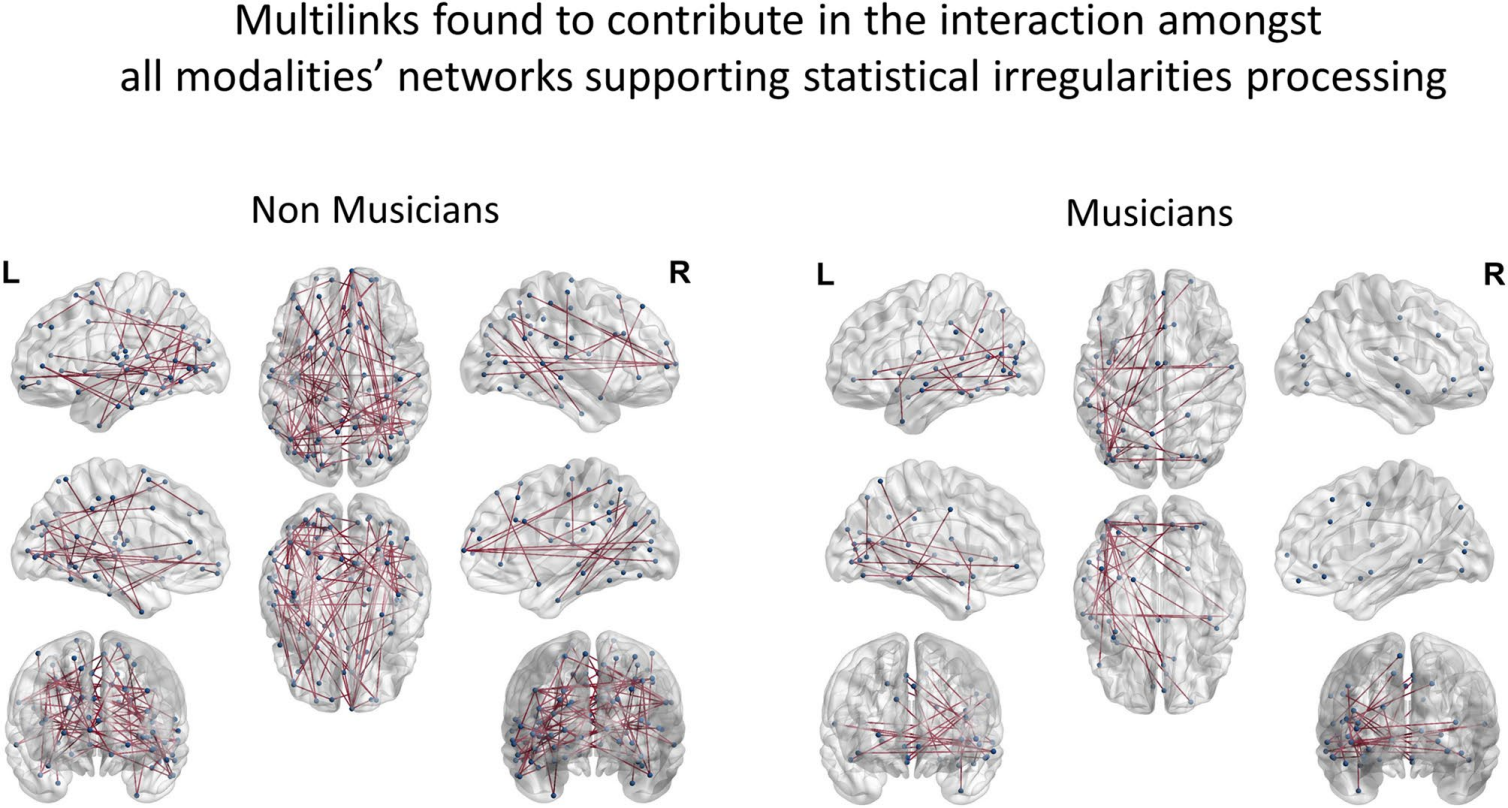
Supramodal mechanism of statistical learning: Regions underlying the interaction amongst all modality-specific networks subserving the identification of stimuli violating transitional probabilities for non-musicians and musicians.

The one-sample t-test estimating within- and between- states connectivity of the group of non-musicians resulted in a significant graph described in Table 1. Overall, in the group of non-musicians, 47 multilinks regions were found to contribute in the interaction amongst all modality-specific networks of irregularity identification (Fig. 3), implying a supra-modal mechanism of statistical deviance detection (names of the regions provided in supplementary file).

**Table 1:** Number (count) of significant (*p* < 0.001) within and between networks multilinks in the different groups as resulted from the one-sample t-test estimating within- and between- states connectivity of each group.

Non-musicians					
		Standard	Audiovisual incongruent	Auditory deviant	Visual deviant
Standard	Edges	175	466	1032	428
	Nodes	150	304	354	286
	Strongest Node	43 right	23c right	47 s right	47 m left
Audiovisual incongruent	Edges		149	601	281
	Nodes		117	316	242
	Strongest Node		44 right	47 s right	23c right
Auditory deviant	Edges			188	509
	Nodes			154	295
	Strongest Node			47 m left	24dd left
Visual deviant	Edges				183
	Nodes				159
	Strongest Node				47 m left

Musicians					
		Standard	Audiovisual incongruent	Auditory deviant	Visual deviant
Standard	Edges	735	216	165	172
	Nodes	313	205	180	182
	Strongest Node	43 right	24dv left	23d	13 l right
Audiovisual incongruent	Edges		701	233	259
	Nodes		299	207	221
	Strongest Node		43 left	23c left	2 right
Auditory deviant	Edges			741	380
	Nodes			307	284
	Strongest Node			45 left	31pd left
Visual deviant	Edges				684
	Nodes				293
	Strongest Node				46 left

**Figure 4:**
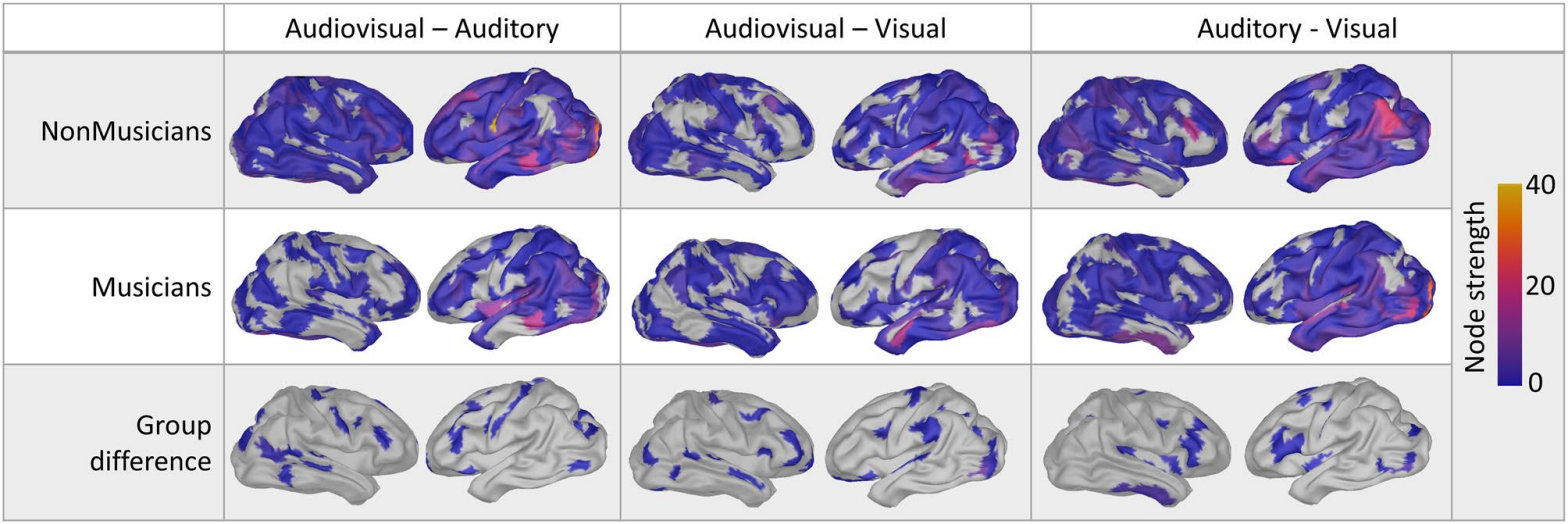
Node degree of between task-defined state of functional connectivity amongst the different sensory modalities and music related neuroplasticity: Node degree of the between-network connectivity projected on an average cortex, presented in a tabular form. First row contains results of non-musicians for each pair of modalities involved in the identification of statistical irregularities (i.e., audiovisual—auditory, audiovisual—visua, auditory-visual), second row presents results of musicians, while the third row presents the 2 × 2 interaction of condition × group, and hence, the effect of expertise related re-organization, for each pair of modalities. The color scale indicates node degree: number of multilinks (edge-to-edge correlation amongst the edges of the network of each independent modality) that involve these cortical regions.

**Figure 5:**
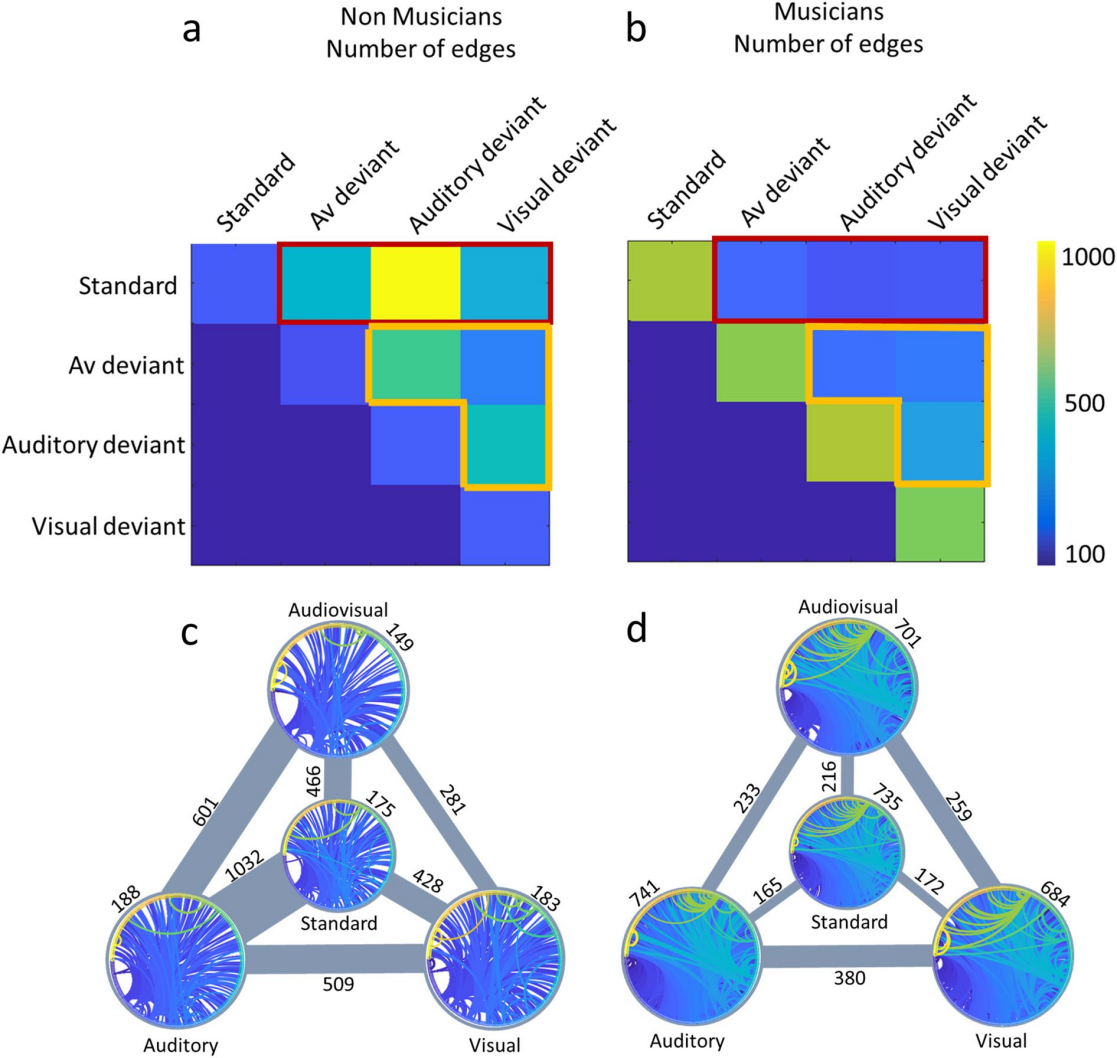
Functional connectivity amongst the different sensory modalities and music related neuroplasticity: task-defined networks depicting inter- and intra- modal functional connectivity for musicians and non-musicians. (**a**): Adjacency matrix representing the graph of non-musicians, showing number of edges via color scaling for each modality and their interactions. (**b**): Adjacency matrix representing the graph of musicians, showing number of edges via color scaling for each modality and their interactions. (**c**): Within and between task-defined network state connectivity of non-musicians. Number of edges between states is represented via line’s size, number of edges within states is given by a circular representation of the network. (**d**): Within and between task-defined network state connectivity of musicians. Number of edges between states is represented via line’s size, number of edges within tates is given by a circular representation of the network.

Node degree of the between-networks connectivity, portraying the cortical areas contributing to the interaction of the different network states, as well as each area’s functional significance in the corresponding multimodal interaction, is depicted in Fig. 4, while the results are summarized in Fig. 5. The significance level for all above-mentioned analyses was set to *p* < 0.001 corrected for multiple comparisons via false discovery rate (FDR) correction, with 10.000 permutations.

The one-sample t-test estimating within- and between- layers connectivity of the group of musicians also resulted in a significant graph, presented in Table 1. Overall, in the group of musicians, 15 multilinks were found to contribute to the interaction amongst all networks, implying a re-organization of the corresponding supra-modal mechanism with a smaller functional role than in the case of non-musicians (Fig. 3).

Node degree of the between-layers connectivity is depicted in Figs. 4 and 5. The significance level for all above mentioned analyses was set to *p* < 0.001 corrected for multiple comparisons via false discovery rate (FDR) correction, with 10.000 permutations.

### Expertise related re-organization of within- and between- network connectivity

**Figure 6:**
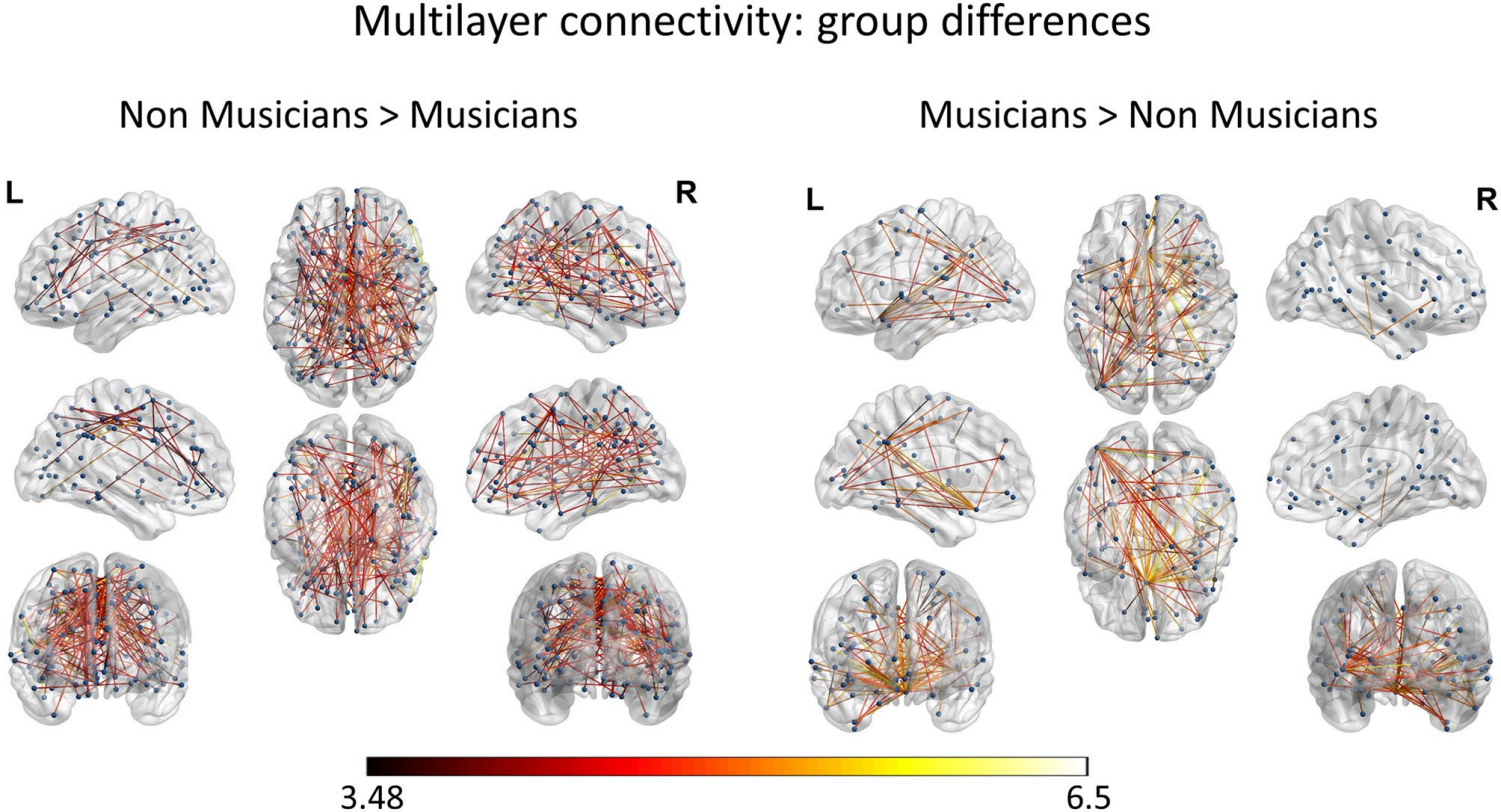
Between groups differences in multilayer connectivity. Statistical parametric maps of the significant networks for the contrast of Non-musicians > Musicians and Musicians > Non-musicians. The color scale indicates t-values, while node strength is represented by node size. Networks presented are significant at *p* < 0.001, FDR corrected, using 10.000 permutations.

The independent samples t-test estimating within- and between- network connectivity differences amongst musicians and non-musicians also resulted in a significant graph. Specifically, the contrast estimating significantly greater within- and between- network connectivity in non-musicians (than musicians) showed a widely distributed network with 192 edges and 267 nodes; and the node with the highest degree was SCEF (SMA) left. On the other hand, the contrast estimating greater within- and between- network connectivity in the group of musicians (than the group of non-musicians) showed a more lateralized network including 106 edges and 123 nodes mostly clustered around area 25 left and temporo-parieto-occipital junction. The results are summarized in Fig. 6. The significance level for all above-mentioned analyses was set to *p* < 0.001 corrected for multiple comparisons via false discovery rate (FDR) correction, with 10.000 permutations. A catalog of all nodes participating in each state of the network is presented in supplementary Table 1.

### Behavioral effects of statistical learning

To evaluate whether statistical learning was reflected behaviorally, we asked our participants, after the exposure to the statistical learning multisensory stream, to respond to a surprise 2-alternative-forced-choice test, in which each regular stimulus pattern was paired with an irregular one. The subjects were instructed to identify which pattern they were more familiar with, estimating whether they could distinguish between patterns respecting the corresponding regularities or the patterns violating them.

The logit transformed percentage of identification of correct patterns, when paired to each modality’s deviant pattern, was compared to the chance level (i.e., the logit transformed chance level is 0) via a one-sample t-test for each group. Both groups successfully used transitional probabilities embedded in the 3 independent modalities’ streams to identify incorrect patterns, showing significant differences from the corresponding chance level. The results of this analysis are presented in Table 2. The mixed model ANOVA with between-subjects factor Group and within-subjects factor Modality, used to evaluate whether musicians and non-musicians differed in their behavioral responses for the 3 different modalities, showed no significant interaction of Group × Modality [F(2, 46) = 0.1; *p* > 0.05; η^2^ = 0.004] indicating that the responses in the different modalities of the two groups did not differ significantly. Hence, the responses to the correct patterns, which respected all different modalities’ regularities, were then used to evaluate via an independent-samples t-test whether, in total, the two groups differed in their statistical learning ability. The result of this analysis [*t*(23) = − 2.815, *p* < 0.05; Hedges’ g effect size = − 1.0930405] revealed that musicians (mean correct responses after logit transformation: 0.979; SD = 0.484) scored significantly higher than non-musicians (mean correct responses after logit transformation: 0.476; SD = 0.406), indicating that musicians’ expertise is related to increased behavioral accuracy in the identification of multisensory irregularities.

**Table 2:** Behavioral results: Logit transformed percentage of identification of correct patterns and comparison to the chance level via a one-sample t-test for each group.

	Musicians	Non musicians
**Audiovisual irregularity**		
Mean	2.22	2.1
SD	0.99	0.54
t-value (Cohen’s d)	11.552 (3.33)	14.267 (3.95)
**Auditory irregularity**		
Mean	2.29	2.0
SD	0.71	0.55
t-value (Cohen’s d)	11.706 (3.37)	13.54 (3.74)
**Visual irregularity**		
Mean	1.95	1.8
SD	0.77	0.52
t-value (Cohen’s d)	8.545 (2.467)	12.617 (3.498)

## Discussion

In the present study, we quantified the inter- and intra- modal interaction of cortical networks integrating the processing of multisensory statistical learning irregularities in musicians and non-musicians. First, we identified brain networks supporting the processing of modality-specific transitional irregularities within the multisensory stream. These networks were significantly re-organized due to music expertise, reflecting an increased involvement of frontal regions in musicians compared to non-musicians. We then assessed the multilayer graph depicting functional connectivity within- and between- task defined network layers. Between tasks connectivity of the group of musicians echoed the conceptual difference between the processing of patterns respecting the transitional probabilities of the stream and all kinds of patterns that violated the corresponding probabilities, a finding that was not present in the group of non-musicians. Additionally, musicians showed greater connectivity within the layers identifying modality-specific irregularities in a multisensory stream and smaller connectivity between the different networks compared to non-musicians, indicating a compartmentalization of the global functionality of statistical learning. Significant differences between the groups in the inter- and intra- modal connectivity were more randomly distributed in the group of non-musicians, while they were more clustered around the anterior cingulate and temporo-parieto-occipital junction in the group of musicians. We argue that this analysis approach can expand our interpretational tools and deepen our understanding of the link between multilayered brain functionality and the hierarchy of higher cognitive processes, such as multisensory integration and neuroplasticity.

Statistical learning is a core process of our higher cognition. It allows our mind to infer the predictability of the incoming sensory input and make sense of a complex multisensory world based merely on exposure^25^. This is grounded on an implicit identification of transitional probabilities that feeds the construction of a generative model describing the underlying structure of the stimulation stream’s regularity. This model is utilized to identify irregularities in the stimulus patterns and applies predictive coding principles^26^ to modify the neuronal response to stimuli violating the corresponding transitional probabilities^27^. Thereby it grounds error processing within the framework of implicit learning^28^. It is no surprise that such a mechanism has been found to act in all sensory modalities^22,29,30^ and across the levels of our cognition’s hierarchy^31^. This fueled the debate about whether statistical learning is a domain-general or domain-specific mechanism^30,32^. The results of the present analysis quantified the neuronal phase interactions between functionally defined states of the network, which represented the processing of stimuli that respected or violated modality-specific transitional probabilities within a multisensory stream. This quantification depicted a stronger correlation amongst the deviance detection networks of the different modalities in comparison to the standard ones. Specifically, 88 common multilinks were found to show correlated connectivity pattern amongst all deviance detection networks in the group of musicians, and 195 in the group of non-musicians, while only 8 common links were found between standard and deviant networks in the group of musicians and 118 in the group of non-musicians. This finding suggests that a common mechanism underlies error identification across the different modalities, distinct from the processing of stimuli that respected statistical regularities. The regions that contributed to this network (Fig. 3) modeled the corresponding supramodal mechanism corroborating the thesis that this mechanism is compartmentalized and differentially applied to the processing of uni-modal stimuli, respecting the requirements of each modality^19^.

Amongst the regions with the most prominent role in our results are several located in the inferior frontal gyrus, (such as BA44 and BA45) and especially region 47 m left, which seems to be involved in all layers of the networks, both for musicians and non-musicians. Additionally, it is the node with the greatest node degree in 5 of the network layers, indicating that amongst the different functionalities that this region serves in learning structured material, such as linguistic or musical syntax^33^, it also has a prominent role in statistical learning, as also indicated by previous fMRI studies in the field^34,35^. Interestingly, this region is argued to show a supra-modal involvement in attention, working memory, and executive functions^36^, while it seems to play a role in decoding complex naturalistic scenes to attend to relevant auditory information^37^, a functionality that is inherent in the paradigm of the present paper.

The comparison, between musicians and non-musicians, of the global architecture of the networks showed that the compartmentalization of this mechanism is re-organized due to expertise^12,38^. Specifically, the functional architecture of the interactions across the states of the networks when identifying modality-specific irregularities within a multisensory stream, as depicted by between states’ number of edges, showed that musicians had, in total, greater compartmentalization of this mechanism. Hence, it seems that non-musicians rely more on the supramodal mechanism to identify statistical irregularities and therefore show greater phase interaction across the states that represent modality-specific deviance detection. In contrast, musicians rely primarily on the processing of modality-specific statistical irregularities, showing smaller connectivity between the different states. This is supported by the fact that a smaller set of regions subserves network interaction amongst all states of the networks in comparison to non-musicians. The smaller contribution of a supramodal mechanism in the group of musicians is coherent with studies showing that musicians have a different neurophysiological respondence and an enhanced implicit learning ability, within the auditory or the audiovisual modality^22,39,40^.

This kind of process, i.e., the enhanced compartmentalization of a supramodal mechanism due to musicianship, seems to serve principles of the free energy principle^41^: expertise strengthens the borders between modality-specific processing and minimizes free energy traveling across the entire system. Hence it seems to optimize connectivity within the stimulus modality, probably by employing neuronal Darwinism principles^42^, and encodes statistical regularities that produce a prediction model more efficiently (as shown by the behavioral results). The denser clustering of the inter-modal network of musicians (see Fig. 5) corroborates this finding. The regions that seem to serve for this clustering (temporo-occipito-parietal junction and anterior cingulate cortex) are areas that have been previously linked with successful statistical learning^43^. Hence, expertise-related neuroplasticity is induced by enhancing the specificity within the system and reducing neuronal synchronization between the network states processing different conditions, reflecting a “fine-tuning” of modality-specific functionality^44^.

The analytical approach followed allowed the modeling of the expertise-related re-organization of the global architecture of multisensory processing. Specifically, our results imply that musicians impose a more significant role for lower-level unisensory auditory and visual interactions to perform multisensory tasks, based on the number of multilinks connecting the auditory and visual networks, than non-musicians do. On the contrary, musicians seem to ground their multisensory integration mainly on the interaction of the auditory with the audiovisual network, hence relying more on higher order processing.

Musicians undergo a great amount of training, during which they integrate auditory, visual, and motor information^45^. This results in an enhancement of auditory^46^ and audiovisual^47^ cortical representations, which are reflected both structurally^48^ as well as functionally^49^. Our results indicate that the cognitive advantage gained from this neuroplasticity process seems to be corroborated by a re-organization of the architecture of the processing hierarch of the cognitive system, which compartmentalizes supra-modal functionalities to a greater degree. This may be interpreted as an attempt from the expert system to free up resources for higher level processing. We must note, though, that the results of the present study do not imply a causal relationship between music training and the corresponding network re-organization, as predisposition effects cannot be ruled out in cross-sectional studies. Nonetheless, such re-organization has been previously described qualitatively and linked to musically induced perceptual changes related to audiovisual processing^49,50^, but our approach quantified this characteristic and allowed its detailed modeling. Thereby, the global weight of each functionally defined process in the corresponding hierarchy of the cognitive system is quantitatively depicted.

Our results, additionally, reveal that several typical multisensory regions have a distinct role in serving the interaction of the different networks. Specifically, the left frontal pole (10v left) seems to support the interaction of uni-sensory auditory and visual networks: In our results, it was identified as a significant interaction node in the corresponding condition, both for the group of musicians as well as for the group of non-musicians, but it was absent in the interaction the other two conditions. This role is in line with the known functionality of this region in integrating results of two or more separate cognitive operations^51^ that are executed to accomplish a single behavioral goal. On the other hand, the left middle temporal gyrus (area MT left) seems to have a greater role in the multisensory integration when audiovisual information is processed and smaller importance when uni-sensory auditory and visual networks interact. This functional distinction may explain their inconsistent appearance in audiovisual studies, depending on the characteristics of the experimental paradigm employed^13,52^ and the stimulus relevance^53^ and may serve to perform predictions based on our results. Noteworthy, indirect interactions between nodes identified via PTE would not alter the functional role that the corresponding regions would have in the inter-network communication; hence such interactions would not influence the corresponding interpretation.

## Conclusion

The present analysis of Phase Transfer Entropy in the MEG data allowed the modeling of the hierarchy of multisensory integration. Between task-defined states of the corresponding networks, functional connectivity revealed a supramodal mechanism supporting the identification of statistical irregularities in stimulation streams, that is compartmentalized and applied, respecting restrictions determined by networks identifying modality-specific irregularities within a multisensory stream. We further show that expertise related re-organization of the global network characteristics, respecting the principles of free energy principle, increased this compartmentalization. Musicians showed greater within- and smaller between network interaction than non-musicians (which showed greater between- and smaller within- network interaction), indicating that non-musicians rely mostly on the identified supramodal mechanism to perform this functionality, while musicians employ mostly the networks supporting statistical deviance detection in a uni-sensory mode. Thus, expertise is linked with an optimization of the global, multilayered structure of the neural network, which can then be applied also for new learning. This results in performing higher cognitive functions with greater efficiency, economy, and smaller amount of system noise. Our findings demonstrate that this methodological approach is conceptually closer to the highly hierarchical structure of brain functionality and can link it successfully to the complexity of our cognitive system.

## Materials and methods

### Subjects

The data used in this study have been described in a previous paper^22^, which employed a different analysis and interpretational approach. The sample of the present study consisted of 25 subjects, 12 musicians, and 13 non-musicians. Musicians (mean age = 26.33; SD = 4.0; 4 males) were retrieved from a pool of students of the Music Conservatory in Münster (mean musical training = 15.47; SD = 3.72). Non-musicians (mean age = 26.7; SD = 5.62; 4 males) had no formal musical education apart from compulsory school lessons. All subjects had normal hearing as evaluated by clinical audiometry, were right-handed according to the Edinburgh Handedness Inventory^54^. Subjects provided informed consent in written form prior to their participation in the study. The study was conducted according to the Declaration of Helsinki, while the protocol was approved by the ethics committee of the Medical Faculty of the University of Münster.

### Stimuli

Stimulation followed a multifeatured oddball paradigm^55^, incorporating multisensory stimuli with uni- and multi-sensory deviants, following the setup of a typical statistical learning procedure^22^. This paradigm employed multilayered stimuli of audiovisual nature, which consisted of 4 distinct characteristics: shape, color, pitch, and timbre. The stimuli were accumulated to build up 4 concurrent but independent and parallel stimulation streams, which were presented simultaneously (hence, all stimulation was of audiovisual nature): an auditory stream, which followed distinct transitional probabilities based on timbral information, a visual, which followed distinct transitional probabilities based on color information, and an audiovisual which was based on the combination of pitch and color information. Respecting the structure of a statistical learning paradigm^56^, the auditory information stream included 11 different timbres, each one related to a specific pitch (i.e., reflective string; pop flute; tenor sax; grand piano; fingerstyle bass; future flute; swirling piano; smokey clav; pop organ; hollywood strings; electric tremolo). The visual stream included 11 different colors, each belonging to a shape. The audiovisual stream was built by a combination of 11 tones (48 kHz, 16bit; pitches: C, C#, D, D#, E, F, F#, G, G# A, B), coupled with 11 different arbitrary complex shapes. The shapes used were adapted from Fiser and Aslin (2001). The Inter Stimulus Interval (ISI) was 150 ms and it was embedded within each pattern, as well as across the patterns. Presentation of the visual stimuli was synchronized with the auditory ones: the duration of visual stimuli was equal to the auditory ones, with a black screen was embedded between the stimuli. The visual stimuli were presented against a black background. All sounds were generated by GarageBand (version 2.2; Apple inc.) while an Attack-Decay-Sustain-Release (ADSR) Envelope was applied, producing a sound with a duration of 400 and 30 ms rise and decay time. Loudness was controlled via the Peak Loudness normalization method, as applied by WavePad Sound Editor (version 7.12; NCH software). The different timbres included: reflective string; pop flute; tenor sax; grand piano; fingerstyle bass; future flute; swirling piano; smokey clav; pop organ; hollywood strings; electric tremolo. The following RGB values were used for the different colors of the visual stimuli: (192, 0, 0; 160, 81,16; 132, 140, 142; 255, 192, 0; 175, 170, 105; 0, 176, 80; 70, 181, 211; 173, 173, 219; 0, 32, 96; 112, 48, 160; 127, 127, 127). All shapes had the same maximum height and width and were presented in the center of a black background.

The stimulation included 4 different categories of patterns interleaved in a continuous stream, building a multifeature regularity (standard, auditory deviant, visual deviant, audiovisual deviant). Each category included 6 different patterns. Each pattern of the first category, serving as standard, included 3 stimuli incorporating the correct associations between shape, color, pitch, and timbre while respecting the statistical regularities of stimulus patterns of all independent streams (i.e., audiovisual, auditory, and visual). Each pattern of the second category, serving as auditory deviant, included 3 stimuli, each incorporating the correct associations between color, pitch, and shape, being standard with respect to the transitional probabilities of the audiovisual and visual stream, while the last of the 3 stimuli had incorrect timbre, producing an auditory deviant. The timbral deviancy of the last tone was based on the use of the timbre that typically belonged to the first stimulus of another standard pattern. Each pattern of the third category, serving as visual deviant, included 3 stimuli, each incorporating the correct associations between timbre, shape, and pitch, while the last of the 3 stimuli had incorrect color, violating the statistical regularities of the visual stream. The color deviancy of the last stimulus was based on the use of the color that typically belonged to the first stimulus of another standard pattern. Each pattern of the fourth category, serving as audiovisual incongruent, included 3 stimuli, each incorporating the correct associations between color and timbre, being standard with respect to the transitional probabilities of the auditory and visual stream, while the last of the 3 stimuli had an incorrect association of shape and pitch, being incongruent with respect to the statistical regularity of the audiovisual stream only. The shape and pitch deviancies were based on the use of the shape and pitch that typically belonged to the first stimulus of another standard pattern. A depiction of the stimulus pattern is presented in Fig. 1.

The presentation paradigm consisted of 3 phases. The initial phase, which followed the requirements of a statistical learning procedure, included 70 patterns from the standard category. The role of this phase was to establish the representations of the underlying structure of the upcoming regularities and had a duration of 1.94 min. The transitional probabilities in this phase were within a range of 0.31–1. The second phase included the 4 different stimulus pattern categories (i.e., standard, audiovisual incongruent, auditory deviant, visual deviant) equally and randomly interleaved in a multisensory oddball paradigm. The transitional probabilities [probability of sequential occurrence between two stimuli^56^] in this phase ranged from 0.23 to 0.75 for the standards and from 0.07 to 0.25 for the deviants. The subjects were exposed to 3 runs of this phase each one lasting 9.5 min and consisting of 460 patterns. Prior to their exposure to the stimulation, subjects were informed of these two phases with the instruction to try to keep their attention to the presented stimuli.

The third phase of the experimental paradigm consisted of a 2-alternative-forced-choice (2AFC) surprise behavioral task. This was performed in the same room as the MEG measurement immediately after the MEG recording. The test contained 36 pairs of patterns, while each pair included one standard and one deviant pattern. The sensory modality of the deviant pattern (i.e., auditory, visual, and audiovisual) was counterbalanced, having, thus, 12 trials for each modality. The patterns within each pair were presented with 300 ms silence in between, while the inter-trial interval was 3 s. The order of the standard and deviant pattern within each pair was counterbalanced. Subjects were asked to indicate which of the two patterns included in each pair were more familiar to them via button presses.

### MRI protocol

T1- weighted MR images from each individual were obtained prior to the experiment, in a 3-Tesla scanner (Gyroscan Intera T30, Philips, Amsterdam, Netherlands). Four hundred contiguous T1-weighted slices of 0.5 mm thickness in the sagittal plane (TR = 7.33.64 ms, TE = 3.31 ms) were collected by a Turbo Field Echo acquisition protocol. The field of view was set to 300 × 300 mm with an in-plane matrix of 512 × 512 setting defining the native voxel size at 0.5 × 0.58 × 0.58 mm^3^. Intensity bias of the images was regularized using SPM8 (Statistical Parametric Mapping, http://www.fil.ion.ucl.ac.uk) to account for differences within each tissue. The images were resliced to isotropic voxels of 1.17 × 1.17 × 1.17 mm. Those images were used to construct Individualized Boundary Element Models (BEM), which were employed in all subsequent forward and inverse problem calculations.

### MEG recordings – instrumentation

Evoked magnetic fields were recorded in a magnetically shielded room via a 275 channel whole-head system (OMEGA, CTF Systems Inc, Port Coquitlam, Canada). Data were acquired continuously using a sampling rate of 1200 Hz. Subjects were oriented in an upright position, while their head was comfortably stabilized inside the MEG dewar using pads. Three head position coils were placed at the nasion, left preauricular, and right preauricular points as fiducial markers, tracking the head position relative to the MEG sensors. The visual stimuli were projected onto the back of a semi-transparent screen positioned approximately 90 cm in front of the subjects’ nasion. An Optoma EP783S DLP projector was used for the projection with a refresh rate of 60 Hz. The viewing angle ranged from − 1.15 to 1.15° in the vertical direction and from − 3.86 to 3.86° in the horizontal direction. Auditory stimuli were delivered via 60 cm long silicon tubes at 60 dB SL above the individual hearing threshold. The latter was determined with an accuracy of 5 dB at the beginning of the procedure for each ear. The subject’s compliance and alertness were ensured by video monitoring. The subjects listened to the 3 stimulation presentation blocks corresponding to the second paradigm phase, with short breaks in between.

### Behavioral data analysis

The behavioral evaluation of statistical learning, as evidenced by the 2AFC task, was based on subjects’ percentage of correct and incorrect responses for each modality and each trial. The responses to a 2AFC task are based on binomial distribution; therefore, to respect the assumptions of parametric tests, their values were transformed via a logit transformation^58,59^ prior to the application of statistical analyses. Initially, a one-sample t-test was performed for each group, comparing the responses to the chance level, to identify whether statistical learning was reflected in the behavior. To evaluate whether the behavioral effects of the different modalities were differentiated across groups, a mixed model ANOVA was used with modality as the within-subjects factor (audiovisual, auditory, and visual) and group as the between-subjects factor (musicians and non-musicians). The analyses were performed using SPSS 25 software (SPSS Inc., Chicago, IL, USA) and the significance level was set to *p* < 0.05.

### MEG data analysis

#### MEG Source activity estimation

Preprocessing of the MEG data was performed using Brainstorm^60^, which is documented and freely available for download online under the GNU general public license (http://neuroimage.usc.edu/brainstorm). Initially, the individual MRI was segmented on 4 different head tissues (i.e., scalp, skull, cerebrospinal fluid (CSF), and brain) using the Computational Anatomy Toolbox (CAT12) for each participant. The latter was subsequently normalized to MNI space via an affine registration using SPM^61^ mutual information algorithm (maff8). A boundary element head model was then generated and used as a volume conductor model for all forward and inverse calculations. The individual positions of MEG sensors were coregistered via anatomical landmarks (nasion, left and right preauricular points) to each subject’s structural MRI.

The 3 runs of continuous functional data were imported and processed separately to ensure that any head movement between the runs would not affect the final outcome. Data were inspected visually to verify quality and event markers were defined. DC offset was removed from the continuous data, but no other filter was applied to avoid temporal smearing of the phase information (restriction of the analysis in the frequency range of 2–40 was performed via a Hilbert-filter, see section “Functional connectivity estimation”). Artifacts due to eye blinks were defined via visual inspection and attenuated via Signal Space Projection. Projectors were defined using principal component analysis (PCA) of the corresponding data segments, while the components best describing the artifacts’ topography were selected manually. Any other bad segments were identified and marked by an automatic artifact detection algorithm implemented by Brainstorm (parts of the signal containing signals larger than 1.2 pT) and were excluded from further analysis. The recorded data were separated into epochs of 1600 ms around the onset of the final stimulus of each triplet, including a pre-stimulus interval of 800 ms, and imported into the database. The specific time-window was chosen to ensure that the epochs include sufficient signal in all frequencies above 4 Hz in the time-window of 0–400 ms which has been shown to include deviance detection responses in statistical learning^29^. DC offset was removed and 4 different event categories were defined: standard, auditory deviant, visual deviant, and audiovisual incongruent. Each epoch was synchronized to the last stimulus of each stimulus pattern. Epochs were baseline corrected using the interval from − 100 to 0 ms, which was free of any stimulation.

**Figure 7:**
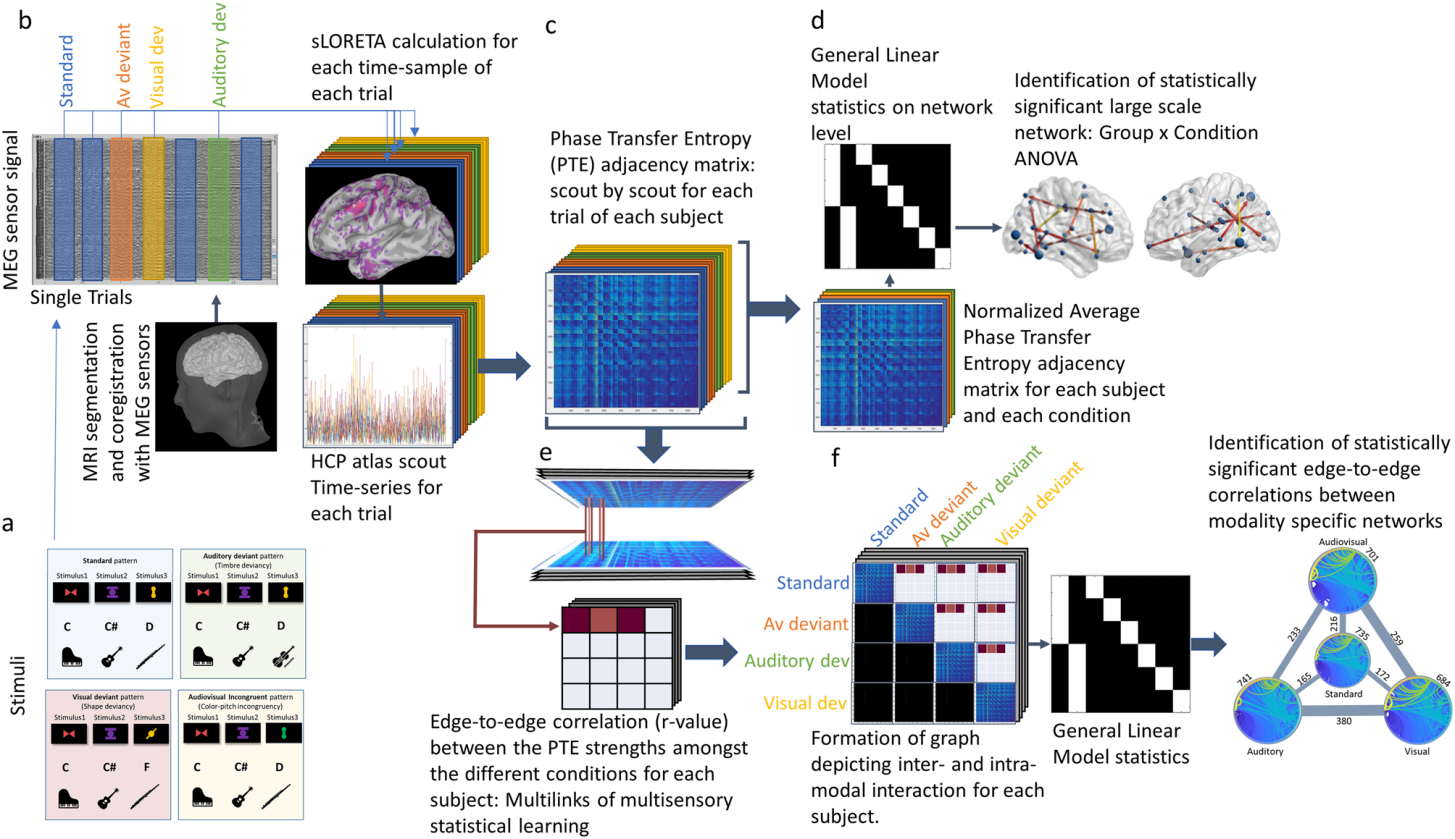
Description of the study’s design: (**A**): A multifeatured multisensory statistical learning stimulation paradigm was applied to 12 musicians and 13 non-musicians. Conditions were: audiovisual standard, audiovisual deviant, auditory deviant, visual deviant. (**B**): MEG data were recorded and analyzed in single trial using an individual head model. Source activity of the 360 regions included in the HCP atlas was calculated via sLORETA for each sample point. (**C**): Phase Transfer Entropy (PTE) amongst the 360 source activities was estimated to generate an adjacency matrix for each trial. (**D**): PTE values of each trial were separately averaged and normalized for each condition, compared via a General Linear Model approach to generate the networks supporting statistical irregularity identification separately for the auditory, visual and audiovisual modalities. Significance was set to *p* < 0.05, corrected for multiple comparisons via FDR, using 10.000 permutations. (**E**): Inter- and intra modal network formation: Calculation of edge-to-edge correlation between the PTE values of the different conditions for each subject. Non-significant correlations were thresholded. (**F**): Formation of a multilayer graph for each subject, representing both between and within task-defined network state connectivity. General Linear Model approach was used for the statistical comparison of the corresponding graphs, depicting between and within modality-specific network connectivity. Significance was set to *p* < 0.05, corrected for multiple comparisons via FDR, using 10.000 permutations.

MEG source analysis was performed on the neural responses of each subject, for each stimulus category, and each single trial to ensure intact phase representation of the signal and for each sample, using constrained sLORETA^62^. sLORETA has been previously used successfully for the estimation of cortical networks supporting audiovisual incongruences^49^ and has the advantage of not needing an a priori definition of the number of activated sources. The resulting source waveforms were downsampled to the 360 sources of the HCP atlas^24^ by choosing the first mode of the principal component analysis decomposition of the signals of each parcel. The specific atlas was selected to provide a balance between (a) sufficient functional and interpretational detail of the reconstruction of cortical activity time-series, as ensured by the multi-modal parcellation, and (b) sufficient reduction of the number of sources reconstructed (Fig. 7a). Finally, the 360 source time-series were extracted.

#### Functional connectivity estimation

The 360 (scouts) × 1921 (samples) time-series of an equal number of trials from each modality (auditory, visual, audiovisual, and standard) were exported to estimate the corresponding within layer functional connectivity matrices. The Phase transfer entropy (PTE)^63^ function for MATLAB (The MathWorks Inc., Natick, MA, US) as implemented by^64^ was used for the estimation of the 360 × 360 adjacency matrix of each trial. PTE is a directed connectivity measure that estimates phase-based information flow (and its direction) based on the transfer entropy between the phase of the time series^65^. The information flow is identified on the basis of a cause-effect relationship demonstrating which region’s signal modulates the signal of (which) other regions. PTE was chosen as a metric that evaluates linear and non-linear phase interactions and their direction, thereby accurately reflecting the primary driver of neuronal synchronization in the framework of multisensory synchronization^13^. The estimation of non-symmetric connectivity matrices grounds the emergence of directional connectivity inferences from the data. PTE is based on nonlinear probability distributions and detects higher-order relations within the signal’s phase information flow, which is a leading source of inter-areal interaction in multisensory perception^66^, while at the same time, it is less affected by source leakage than other TE metrics^63^. The estimation of PTE is performed independently for every edge of the network, minimizing the dependency of the extent of the network tested (number of nodes included) to the amount of the data^63^. The delay of the transfer entropy (i.e., the time delay from the past of both the source as well as target to the next value of the target) was set to 10. Hilbert transformation is used to determine the phases of the signals and restrict the analysis to the frequency range of 2–60 Hz, which is expected to include event related deviance detection responses^67^. The number of time steps of the past of the target (i.e., embedding length k) was set to 1. The bin size for the histograms of phase occurrences was determined following the approach proposed by Nason and Scott 1993, due to the small sample size. The complete frequency spectrum was entered as a single band for the PTE estimation (2–60 Hz), allowing us to directly investigate linear and non-linear interactions amongst the phase oscillations of all frequencies of interest. The directed, and hence asymmetric, adjacency matrices of the different epochs of each subject were then averaged (after the calculation of PTE), resulting in one connectivity matrix per category per subject (Fig. 7b), and normalized via a z-score transformation.

#### Statistical analysis of modality-specific identification of statistical learning irregularities

The Network Based Statistic (NBS)^69^ toolbox was used to identify statistically significant connections using General Linear Model. A non-parametric F-test between the adjacency matrix of each modality’s (auditory, visual, audiovisual) standard and deviant, within each group (musicians, non-musicians) was implemented via NBS to identify the functional connectivity network supporting the modality-specific, statistical learning, error identification of each group. Specifically, a 2 × 2 mixed model analysis for each modality was also implemented via NBS, with between-subjects factor Group and within-subjects factor Condition (Standard, Deviant) to explore, via the Group × Condition interaction, the musical expertise related neuroplastic re-organization of the corresponding functional network. The significance level for both analyses was set to *P* < 0.001 corrected for multiple comparisons via false discovery rate (FDR) correction, while 10.000 permutations were used. Density and node strength of the significant networks, as identified via this analysis, were then estimated using Brain Connectivity Toolbox^70^. The visualization of the significant networks as weighted graphs was performed using BrainNet Viewer^71^ (Fig. 7c).

#### Estimations of interaction within and between task-defined state of the networks and statistical analyses

Estimations of interaction within and between modality-specific networks were based on the estimation of pairs of edges having related connectivity profiles in the different states. Thereby, for each subject, an edge-to-edge Pearson correlation between the PTE values of any two stimulation modalities (standard and auditory deviant, standard and visual deviant, etc.) was estimated, for every single trial, to depict pairs of regions having correlated connectivity profiles when processing these two categories of stimulus patterns^72^. Hence, a 360 × 360 adjacency matrix was constructed, including for each pair of nodes the correlation value between the PTE derived edges connecting the corresponding regions in the two different conditions. Only significant correlations (positive and negative) were entered in the matrix, while all non-significant ones were thresholded. The use of positive and negative correlations was chosen to incorporate a broad definition of whether two edges are “related”^73^. Hence, each connected component in the thresholded edge-edge correlation matrix forms a multilink depicting between network connectivity (Fig. 7d).

The above-described process resulted in 6 adjacency matrices containing the corresponding multilinks for each subject. Then, the adjacency matrix containing the z transformed PTE values of each independent condition, and the adjacency matrices containing the multilinks (that is, the significant correlations between the corresponding connectivity profiles) of all pairs of conditions, were connected to form a square multilayer graph of 1440 × 1440, which is compartmentalized in 4 × 4 sets of 360 nodes, each describing the corresponding network’s edge or the correlation between the edges of these regions in the different conditions (standard, audiovisual, auditory and visual) (Fig. 7e).

The Network Based Statistic (NBS)^69^ toolbox was used to identify statistically significant multisensory networks via a one-sample t-test between the graphs for each group (Fig. 7f). Also, a between groups t-test was utilized to identify significant differences in the graphs of musicians and non-musicians, estimating musical-expertise related neuroplastic effects on the interaction between the different states of the multisensory integration networks. The significance level for all analyses was set to *P* < 0.001 corrected for multiple comparisons via false discovery rate (FDR) correction, while 10.000 permutations were used. As qualitative descriptors of the global network architecture, network density and node degree of the significant multilinks, independently for each set of between task-defined states of the connectivity networks, were then estimated using in-house scripts.

## Supplementary Information

Supplementary Information.

## Data Availability

The EEG datasets analyzed during this study are available at [G-Node] and are accessible via the following link: [https://gin.g-node.org/parasvag/MultisensoryStatisticalLearning.git].
